# Knowledge, adherence and control among patients with hypertension attending a peri-urban primary health care clinic, KwaZulu-Natal

**DOI:** 10.4102/phcfm.v9i1.1456

**Published:** 2017-10-19

**Authors:** Olumuyiwa A. Olowe, Andrew J. Ross

**Affiliations:** 1 Mbogolowane District Hospital, Kwapett, South Africa; 2 School of Nursing and Public Health, College of Health Sciences, University of KwaZulu-Natal, South Africa

## Abstract

**Background:**

Despite hypertension being a common condition among patients attending primary health care (PHC) clinics, blood pressure (BP) control is often poor. Greater insight into patient-related factors that influence the control of hypertension will assist in the development of an intervention to address the issues identified.

**Aim:**

The aim of the study was to assess patient-related variables associated with hypertension control among patients attending a peri-urban PHC clinic.

**Setting:**

The setting for this study was a peri-urban PHC clinic in KwaZulu-Natal.

**Method:**

This was an observational, descriptive and cross-sectional study with 348 patients selected over a 1-month period. A validated questionnaire was used to collect data on patients’ hypertension knowledge and self-reported adherence, and BP recordings from their medical record were recorded to ascertain control.

**Results:**

Of the 348 participants, only 49% had good BP control and 44% (152/348) had concurrent diabetes mellitus. The majority of patients had moderate levels of knowledge on hypertension and exhibited moderate adherence. There was a significant relationship between knowledge and reported adherence, between reported adherence and control, but not between reported knowledge and control.

**Conclusion:**

Despite over 90% of the study population having moderate knowledge, and 62% with moderate reported adherence, BP was well controlled in only less than 50% of the study population. These findings suggest a need to emphasise adherence and explore new ways of approaching adherence.

## Introduction

Developing countries, faced with a high burden of communicable and non-communicable diseases, are struggling to provide adequate health care services to their population.^1^ Hypertension is one of the most prevalent non-communicable diseases worldwide, with an estimated 1 billion afflicted people in 2008,^2^ a number which is projected to increase to 1.56 billion by 2025.^3^ In sub-Saharan Africa (SSA), the number of patients with hypertension is estimated to increase from 74.7 million in 2008 to 125.5 million in 2025, an increase of 68% which will put increased pressure on the health care services.^4^ A systematic review of the evidence on the prevalence and management of hypertension in SSA estimated that < 40% of those with a BP ≥ 140/90 mmHg were diagnosed as having hypertension, < 30% of those diagnosed were on antihypertensive treatment and < 20% of those on treatment had their BP controlled.^4^

In South Africa (SA), it is estimated that a quarter of the population between the ages of 15 and 64 suffer from hypertension, which equates to over 6.2 million people if a BP measurement of ≥ 140/90 mm Hg is used as indicative of hypertension.^5^ Studies on hypertension in SA have found suboptimal BP control among hypertension patients in both rural and urban settings,^6,7,8,9,10,11^ suggesting a vast room for improvement in the diagnosis and management of hypertension.^4^

Hypertension is a complex chronic condition often referred to as a ‘silent killer’ and a key contributor to the development of cardiovascular and cerebrovascular diseases.^2^ The ultimate goal of managing hypertension, like any other non-communicable disease, is to achieve target control and prevent the development of complications. This involves a multipronged approach including education of patients about the causes, management and complications of hypertension. Poor control of hypertension is a major risk for the development of cardiovascular and renal diseases, and a clear, consistent plan at diagnosis has been shown to help prevent complications.^12^ A number of studies have shown that adequate patient knowledge of hypertension (including patient knowledge of their target BP) improves patients’ adherence to medication and improves control of their BP.^11,13,14^ However, this correlation between patients’ knowledge about hypertension and control of their BP is not a consistent finding, with a study at an urban district hospital in Durban in 2013 showing no significant relationship between patients knowing their target BP and actually having a controlled BP.^8^ Reasons for poor control of hypertension are known to be complex and include factors such as patients’ low level of education about hypertension, non-adherence, physician inertia to treat to targets, health system factors, lack of antihypertensive medications and long distances to health facilities, all of which have been reported in many African countries.^4,6,10,15,16,17^

Most of the studies on hypertension have been based in urban or rural areas,^6,7,8,9,10^ with less research among patients with hypertension attending peri-urban PHC clinics.^11^ It is important to study hypertension in peri-urban areas as there are unique factors influencing diagnosis and management of hypertension, including diet,^5,18^ poverty^19^ and unemployment,^20^ which differ from other settings. The aim of this study was to (1) describe the demographic characteristics of a peri-urban PHC-based population of patients with hypertension; (2) assess level of their knowledge of hypertension, reported adherence to antihypertensive medications and BP control; and (3) review whether there were associations between level of education, age of patients, duration of disease, knowledge, reported adherence and BP control. It is hoped that this study will add to the knowledge around hypertension in the peri-urban South African PHC population and will guide the development of strategic interventions to address the issues identified.

## Method

The study design was an observational, descriptive, cross-sectional study conducted at a peri-urban PHC clinic in KwaZulu-Natal. At this clinic, hypertensive patients were mainly cared for by nurses with referral to a doctor for review if the BP was uncontrolled or complications occurred. The catchment population of the clinic was 27 276 people, with an average of 1197 patients with hypertension being treated at the clinic each month. Data were collected between May and June 2015. Inclusion criteria were patients with hypertension above the age of 18 years who had been collecting hypertensive medication from the PHC clinic for at least 1 year. People under 18 years and those not willing to participate were excluded. A sample size of 350 patients was deemed adequate after calculation and discussion with a statistician (95% confidence level and 5% confidence interval).^21^ It was assumed that hypertensive patients present for care at the clinic in a non-systematic order and every third patient meeting the inclusion criteria was invited to partake until the sample size of 348 was reached. Files were marked to ensure that participants were only assessed once.

Data sources were twofold. (1) Clinical information about BP recordings was obtained from patients’ records. (2) Other data were collected during patient interviews by a research assistant who was fluent in English and isiZulu, which was the first language of the majority of patients in this context. The interviews were structured in a closed-ended questionnaire which comprised five sections. Section One captured the socio-demographic data of participants (including variables such as gender, age, educational level, race, employment status, marital status and comorbid chronic disease). Section Two comprised 18 questions sourced and modified from a Hypertension Fact Questionnaire (HFQ) previously validated by Saleem et al.^22^ Saleem et al. studied hypertension in Pakistan, which is a developing-world context with a population of varying levels of literacy who do not speak English as a first language.^22^ Permission to use the questionnaire was obtained from the author before the study. The HFQ considered various variables around hypertension knowledge, with a potential maximum score of 18 and a minimum of 0. A score of < 9 correlated with poor patient knowledge about hypertension, 9–14 moderate knowledge and ≥ 15 good knowledge. In Section Three, participants were asked to identify their medications and dosage. Section Four contained questions modified from the Morisky 8-item medication adherence scale. This scale was developed in 1986 and employs a structured four-item self-reported adherence measure.^23^ The alpha reliability of this adherence scale is 0.61 and literature describes that the scale can be easily integrated into routine medical visits. Items in the scale address barriers to medication-taking and permit the health care provider to reinforce positive adherence behaviours. A score of > 2 indicates low adherence, 1–2 medium adherence and 0 high adherence. In Section Five, the patients’ last three BP readings (including that at the time of data collection) were recorded. Any patient with a BP > 140/90 mmHg was classified as poorly controlled. The questionnaire was pretested in another PHC facility among 15 patients with hypertension in order to revalidate it.

Data were captured and analysed using Epi info version number 7.1.5.2. Results were summarised by frequencies and percentages (for categorical variables). Comparisons between independent variables and outcome variables were carried out using the Fisher’s exact test. A *p*-value of < 0.05 was considered to be statistically significant.

### Ethical consideration

Ethical permission for this study was provided by the Biomedical Research Ethics Committee of the University of KwaZulu-Natal (Ref: 330/14). Permission was also obtained from the KwaZulu-Natal Provincial Department of Health and PHC clinic management team. All patients who participated in the study signed informed consent after being given an information sheet informing them of the purpose of the study.

## Results

A total of 348 patients participated in the study. Results are presented as follows: (1) demographic characteristics, (2) knowledge levels, (3) reported adherence level, (4) BP control and (5) associations between variables.

### Demographic characteristics

Female participants represented the majority of the respondents, with most being black Africans and also married. Just over 40% were in the age group of 51– 60 years with over half having only primary education and < 1% having tertiary education. The majority was unemployed and diabetes mellitus was the commonest co-morbidity. Most participants had been on treatment for hypertension for > 5 years. The demographic characteristics are shown in greater detail in Table 1.

**TABLE 1:** Summary of demographic characteristics (*N* = 348).

Characteristics	Frequency	Percentage (%)
**Gender**		
Male	75	21.6
Female	273	78.5
**Age group (years)**		
21–30	3	0.9
31–40	13	3.7
41–50	65	18.7
51–60	141	40.5
61–70	86	24.7
> 70	40	11.5
**Educational status**		
None	99	28.5
Primary	213	61.2
Secondary	34	9.8
Tertiary	2	0.6
**Race**		
Black people	342	98.3
White people	4	1.2
Indian people	2	0.6
**Employment status**		
Employed	31	8.9
Unemployed	317	91.1
**Marital status**		
Single	94	27.0
Married	253	72.7
Divorced	1	0.3
**Co-morbid chronic conditions**		
Angina	2	0.6
Arthritis	47	13.5
Asthma	19	5.5
Diabetes mellitus	152	43.7
None	100	28.7
Other	28	8.1
**Duration of hypertension (years)**		
< 5	79	22.7
5–9	68	19.5
10–14	78	22.4
15–19	47	13.5
20–24	30	8.6
25–29	18	5.2
≥ 30	28	8.1

### Knowledge of hypertension

Most patients achieved a ‘moderate knowledge-level’ score (314; 90%), with an average knowledge score of 10.36 Table 2 summarises the hypertension knowledge score of participants. Only one patient achieved a ‘good knowledge-level’ score. Participants scored poorly when answering questions relating to (1) normal and target BP values and (2) if medication alone was enough to ensure good BP control. Only one participant correctly mentioned the expected BP targets. Participants scored high around questions relating to lifestyle modification and all participants knew that poor hypertension control could lead to life-threatening complications. Over 95% did not know the names of their antihypertensive medication, whereas 99.7% were able to identify their medication from sample packets presented to them.

**TABLE 2:** Hypertension knowledge score (*N* = 348).

Hypertension Fact Questionnaire score	Knowledge levels	Frequency	Percentage (%)
> 15	Good knowledge	1	0.3
9–14	Moderate knowledge	314	90.2
< 9	Poor knowledge	33	9.5

### Reported adherence

The self-reported adherence level to the antihypertensive medications of the studied population was moderate (218; 63%) – see Table 3 for details. Most participants reported that they forget to take medication sometimes and do not engage in regular prescribed exercise on most days.

**TABLE 3:** Reported adherence levels (*N* = 348).

Morisky Medication Adherence Scale	Reported adherence level	Frequency	Percentage (%)
0	High	17	4.9
1–2	Moderate	218	62.6
> 2	Low	113	32.5

### Recorded blood pressure

Only 49% of participants had optimal-level BP as measured over their last three visits to the PHC clinic.

### Associations between variables

Table 4 highlights the association between variables, with significant association found between participants’ level of education and their knowledge of hypertension (*p* = 0.03), between patients’ education level and their reported adherence level (*p* = 0.01), between knowledge and reported adherence (*p* < 0.00) and between reported adherence, knowledge and BP control (*p* < 0.00).

**TABLE 4:** Associations between independent variables and outcome variables.

Independent variables	Outcome variables and *p*-value		
	Knowledge	Adherence	BP control
Level of education	0.03	0.01	0.61
Age	0.33	0.10	0.83
Duration of disease	0.80	0.62	0.33
Knowledge score	-	0.00	0.30
Adherence score	0.00	-	0.00

BP, blood pressure.

## Discussion

This study provides a snapshot of hypertension knowledge, adherence and control at a peri-urban PHC clinic. The study is consistent with other studies which have demonstrated poor hypertension control,^6,7,8,9,11^ with this study showing that just under half of the population studied had adequate hypertension control. The seriousness of this finding is compounded by the fact that the commonest comorbid condition (152/348; 43.7%) was diabetes mellitus. The importance of good BP control in diabetic patients has been long stressed in medical literature. The study also demonstrated that a few patients were already experiencing the consequences of poorly controlled hypertension, with some patients suffering from angina.

Participants in this study were mainly African and female which is similar to other South African studies on hypertension.^7,8,10^ This finding may be due to better help-seeking behaviour in women than among men.^13^ This finding is of concern as population studies show that men and women are equally affected by hypertension and men are more likely to present with complications associated with hypertension because they do not access care.^13^ Community-based screening programmes, with a specific focus on men may increase the number of men found to have hypertension. The screening could also encompass diabetes screening, and assistance should be available for those who are unemployed (the majority of the study population), as attending a PHC clinic for screening and management may be prohibitively expensive for them.

Most participants in this study had only primary-level education. This has significant implications for control of hypertension in this population; educational materials must be delivered in a form that can be understood by those with lower literacy levels. Educational materials must be available in the first language of most of the participants. Visual education methods may prove useful as most participants could not name their medications but knew what they looked like. Dennison has shown that tailored education is important in increasing patient understanding of hypertension and contributes to improved control of hypertension.^11^

This study showed that the vast majority of participants (314/348; 90.2%) had moderate hypertension knowledge which was an encouraging finding. An association was found between a higher level of education and better hypertension knowledge and better reported adherence. In addition, there was a significant association between knowledge and reported adherence, a finding similar to a study done by Gaili et al.^24^ in the United Arab Emirates. However, it is of concern that there was no association found between knowledge of hypertension and good hypertension control. This finding is consistent with a hypertension study carried out in a district hospital in KwaZulu-Natal, which also found no association between the level of hypertension knowledge (including knowledge about target BP) and hypertension control,^8^ suggesting that knowledge alone is insufficient for good control. This requires further investigation as factors (e.g. access to clinic) may have a more important bearing on hypertension control than patient knowledge and reported adherence.^25^ It is worth noting that adherence was self-reported and the actual adherence may differ significantly from self-reported adherence.

There was no statistically significant association between age group and duration of hypertension with regard to knowledge, adherence and control. It was of concern that increased duration of hypertension was not associated with increased knowledge, adherence to antihypertensive medication or hypertension control as would perhaps be expected. This is in contrast to a study which has shown that with increasing duration of disease, patients’ knowledge about the disease increases through regular and active health education.^14^ This finding requires further investigation.

This study found a significant association between adherence and BP control. This is not an unexpected finding, as adherence to medication should be associated with better BP control. This suggests a need to focus on an approach that promotes adherence as a behaviour change strategy in the control of BP. A study in Pakistan in 2011 demonstrated that continual reinforcement of education and responding to queries around adherence lead to improved, sustained adherence.^22^ In addition, health care providers could consider using the 5As approach, which has been found to be successful in behaviour change counselling (ask, alert, assess, arrange and assist), as a structured, proactive approach to motivate for changes in adherence behaviour.^26^ Improved adherence, in turn, should have a positive effect on hypertension control, with a resultant decrease in complications that can arise from uncontrolled hypertension.^22^ Patients must also be encouraged to play an active role in controlling their hypertension, including asking questions freely at every opportunity, as this has been shown to improve knowledge, adherence and control.^27^ Adherence is key to achieving good control of BP, but with only about 4.9% of the studied population having high adherence levels, more needs to be done to explore the causes of poor adherence and to find pragmatic strategies to address any issues identified.^28^ Unless this is addressed, the low hypertension control rate, especially when associated with concurrent diabetes, will lead to enhanced morbidity and mortality and associated higher expenditure on hypertension-related illnesses.

### Limitations of the study

A limitation of the study is the convenient method of sample selection. An additional limitation is that the data collection instrument considered self-reported adherence, and this was not verified in any way. The study only considered patient-related variables that impact on BP control without considering other factors, including health care-related factors, which could affect BP control.

## Conclusion

It was of concern that half of the study population has less than optimally controlled hypertension and 43% of the study population had concurrent diabetes. The hypertension-knowledge and self-reported adherence rates were found to be only moderate. There is thus an immediate opportunity for improving both knowledge and adherence. However, any intervention must consider the unique characteristics of a peri-urban South African population. Further study is required around the gendered nature of hypertension in this study and most importantly around why hypertension control levels are so low. Research must also move beyond the patients to consider the role of the health care provider, the health care facility and the health care system in promoting greater adherence to antihypertensive medication.
